# *catena*-Poly[[[bis­(4-pyridine­aldoxime-κ*N* ^1^)zinc]-μ-benzene-1,4-dicarboxyl­ato-κ^2^ *O* ^1^:*O* ^4^] 4-pyridine­aldoxime monosolvate]

**DOI:** 10.1107/S1600536813006107

**Published:** 2013-03-16

**Authors:** Hitoshi Kumagai, Satoshi Kawata, Yoshiyuki Sakamoto

**Affiliations:** a Toyota Central R&D Labs., Inc., Nagakute 41-1, Aichi, Japan; b Department of Chemistry, Fukuoka University, Fukuoka 814-0180, Japan

## Abstract

In the title compound, {[Zn(C_8_H_4_O_4_)(C_6_H_6_N_2_O)_2_]·C_6_H_6_N_2_O}_*n*_, the Zn^II^ ion exhibits a tetra­hedral coordination environment defined by two benzene-1,4-dicarboxylate dianions and two 4-pyridinealdoxime ligands. The dianions bridge the Zn^II^ ions, giving a zigzag chain along the *b* axis. Adjacent chains are connected by O—H⋯O hydrogen bonds, forming a cavity in which an uncoordinating 4-pyridine­aldoxime mol­ecule is located; this mol­ecule is linked by O—H⋯O and O—H⋯N hydrogen bonds to the zigzag chain.

## Related literature

For coordination polymers, see: Cheetham *et al.* (1999 ▶); Furukawa *et al.* (2010 ▶). For related host–guest systems, see: Kitagawa & Kawata (2002 ▶); Lehn (1995 ▶).
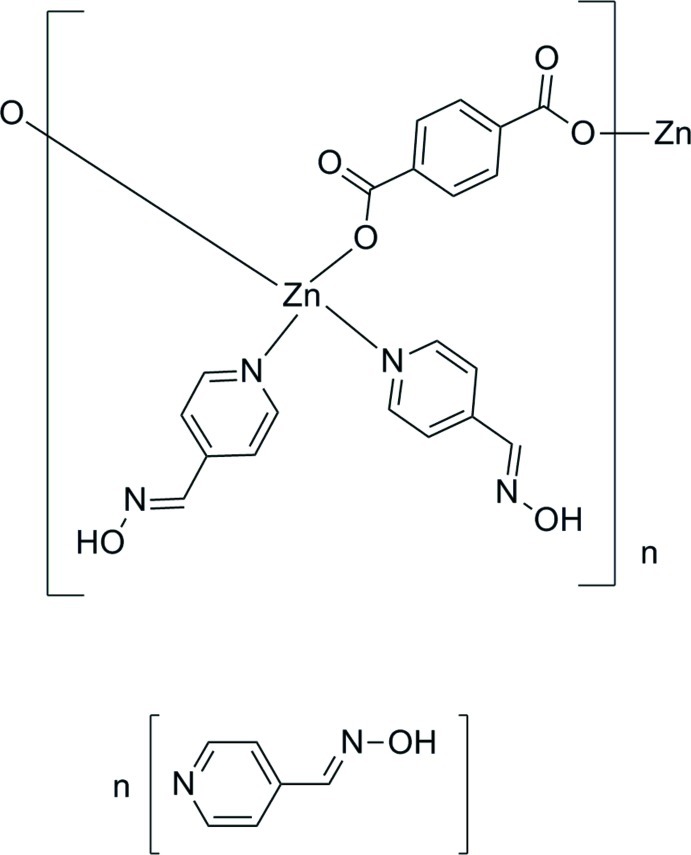


## Experimental

### 

#### Crystal data

[Zn(C_8_H_4_O_4_)(C_6_H_6_N_2_O)_2_]·C_6_H_6_N_2_O
*M* *_r_* = 595.88
Monoclinic, 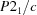

*a* = 7.583 (3) Å
*b* = 15.831 (6) Å
*c* = 21.906 (8) Å
β = 98.516 (3)°
*V* = 2600.7 (16) Å^3^
*Z* = 4
Mo *K*α radiation
μ = 1.00 mm^−1^
*T* = 293 K
0.60 × 0.40 × 0.15 mm

#### Data collection

Rigaku Mercury70 diffractometer
Absorption correction: multi-scan (*REQAB*; Rigaku, 1998 ▶) *T*_min_ = 0.714, *T*_max_ = 0.860
24675 measured reflections
5922 independent reflections
5402 reflections with *I* > 2σ(*I*)
*R*_int_ = 0.019

#### Refinement

*R*[*F* ^2^ > 2σ(*F* ^2^)] = 0.030
*wR*(*F* ^2^) = 0.078
*S* = 1.06
5922 reflections
373 parameters
H atoms treated by a mixture of independent and constrained refinement
Δρ_max_ = 0.34 e Å^−3^
Δρ_min_ = −0.27 e Å^−3^

### 

Data collection: *CrystalClear* (Rigaku/MSC, 2005 ▶); cell refinement: *CrystalClear*; data reduction: *CrystalClear*; program(s) used to solve structure: *SIR2008* (Burla *et al.*, 2007 ▶); program(s) used to refine structure: *SHELXL97* (Sheldrick, 2008 ▶); molecular graphics: *CrystalStructure* (Rigaku, 2010 ▶); software used to prepare material for publication: *CrystalStructure*.

## Supplementary Material

Crystal structure: contains datablock(s) global, I. DOI: 10.1107/S1600536813006107/is5248sup1.cif

Structure factors: contains datablock(s) I. DOI: 10.1107/S1600536813006107/is5248Isup2.hkl

Supplementary material file. DOI: 10.1107/S1600536813006107/is5248Isup3.cdx

Additional supplementary materials:  crystallographic information; 3D view; checkCIF report

## Figures and Tables

**Table 1:** Hydrogen-bond geometry (Å, °)

*D*—H⋯*A*	*D*—H	H⋯*A*	*D*⋯*A*	*D*—H⋯*A*
O5—H3⋯N5^i^	0.89 (5)	1.81 (4)	2.692 (3)	172 (4)
O6—H9⋯O4^ii^	0.81 (3)	1.94 (3)	2.752 (3)	177 (3)
O7—H13⋯O4^i^	0.77 (4)	2.07 (4)	2.800 (3)	158 (4)

Symmetry codes: (i) 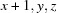; (ii) 
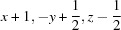
.

**Fig. 1.**
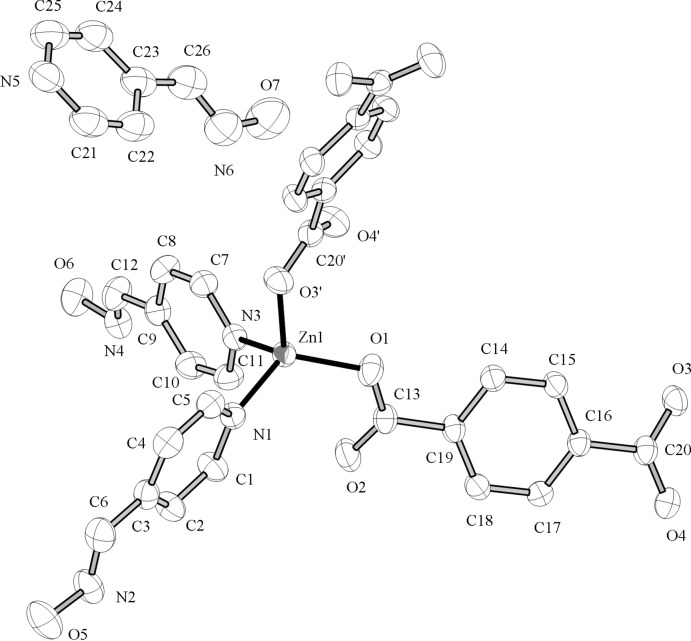
View of the title compound with atomic numbering scheme. Hydrogen atoms are omitted for clarity.

**Fig. 2.**
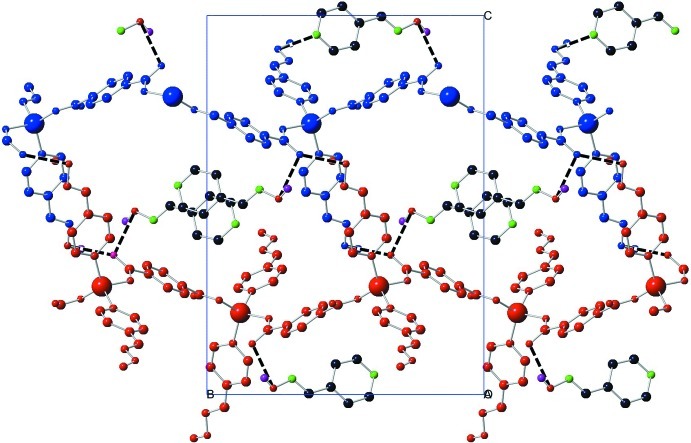
Hydrogen bonding interactions between chains (blue and red) and intercalated 4-pyNOH molecules. Hydrogen atoms and hydrogen bonding interactions are shown as purple color and dashed line, respectively.

## supplementary crystallographic information

### Comment

The design and synthesis of coordination polymers (CPs) have received considerable attention in recent years due to potential applications for magnetic materials, sorption and host–guest materials (Cheetham *et al.*, 1999; Furukawa *et al.*, 2010; Lehn, 1995). Intermolecular interactions, such as coordination bonding, hydrogen bonding and van der Waals forces, have been used to produce such network materials. Among them, hydrogen bonding interaction is an important interaction to realize self-assemblies of molecules with novel properties such as proton-transfer-mediated electron transfer, non-linear optics and thermochromic properties of crystalline forms. We have focused on the synthesis and characterization of one-dimensional coordination polymers as host materials and have found a number of host–guest systems using hydrogen bonding interactions (Kitagawa & Kawata, 2002). Here we report synthesis and single-crystal structure of a new one-dimensional coordination polymer which consists of tetrahedral Zn(II) ion, 1,4-benzenedicarboxylate as a bridging ligand and 4-pyridineoxime (4-pyNOH) as terminal ligands. Two types of 4-pyridinealdoxime are found in the crystal. One is uncoordinated and the other 4-pyNOH coordinates to Zn(II) ion. Uncoordinated 4-pyNOH molecules are stabilized by intermolecular hydrogen bonds in the cavity formed by hydrogen bonds between chains.

### Experimental

An aqueous solution (5 ml) of zinc nitrate hexahydrate (0.29 g) was transferred to a glass tube, then an ethanol-water mixture (5 ml) of terephthalic acid (0.17 g), NaOH (0.08 g) and 4-pyNOH (0.24 g) was poured into the glass tube without mixing the two solutions. Colorless crystals began to form at ambient temperature in 2 months. One of these crystals was used for X-ray crystallography.

### Refinement

Hydrogen atoms bonded to carbon atoms were introduced at the positions calculated theoretically and treated with riding models with *U*_iso_(H) = 1.2*U*_eq_(C). Other hydrogen atoms (H3, H9, H13) were located in a Fourier difference map and refined freely [O—H = 0.77 (4)–0.89 (5) Å].

### Crystal data

**Table:**

[Zn(C_8_H_4_O_4_)(C_6_H_6_N_2_O)_2_]·C_6_H_6_N_2_O	*F*(000) = 1224.00
*M**_r_* = 595.88	*D*_x_ = 1.522 Mg m^−^^3^
Monoclinic, *P*2_1_/*c*	Mo *K*α radiation, λ = 0.71070 Å
Hall symbol: -P 2ybc	Cell parameters from 5655 reflections
*a* = 7.583 (3) Å	θ = 3.0–27.5°
*b* = 15.831 (6) Å	µ = 1.00 mm^−^^1^
*c* = 21.906 (8) Å	*T* = 293 K
β = 98.516 (3)°	Platelet, colorless
*V* = 2600.7 (16) Å^3^	0.60 × 0.40 × 0.15 mm
*Z* = 4	

### Data collection

**Table:**

Rigaku Mercury70 diffractometer	5402 reflections with *F*^2^ > 2σ(*F*^2^)
Detector resolution: 7.314 pixels mm^-1^	*R*_int_ = 0.019
ω scans	θ_max_ = 27.5°
Absorption correction: multi-scan (*REQAB*; Rigaku, 1998)	*h* = −9→9
*T*_min_ = 0.714, *T*_max_ = 0.860	*k* = −20→20
24675 measured reflections	*l* = −28→28
5922 independent reflections	

### Refinement

**Table:**

Refinement on *F*^2^	Secondary atom site location: difference Fourier map
*R*[*F*^2^ > 2σ(*F*^2^)] = 0.030	Hydrogen site location: inferred from neighbouring sites
*wR*(*F*^2^) = 0.078	H atoms treated by a mixture of independent and constrained refinement
*S* = 1.06	*w* = 1/[σ^2^(*F*_o_^2^) + (0.0395*P*)^2^ + 0.8505*P*] where *P* = (*F*_o_^2^ + 2*F*_c_^2^)/3
5922 reflections	(Δ/σ)_max_ = 0.002
373 parameters	Δρ_max_ = 0.34 e Å^−^^3^
0 restraints	Δρ_min_ = −0.27 e Å^−^^3^
Primary atom site location: structure-invariant direct methods	

### Special details

**Table:**

Refinement. Refinement was performed using all reflections. The weighted *R*-factor (*wR*) and goodness of fit (*S*) are based on *F*^2^. *R*-factor (gt) are based on *F*. The threshold expression of *F*^2^ > 2.0 σ(*F*^2^) is used only for calculating *R*-factor (gt).

### Fractional atomic coordinates and isotropic or equivalent isotropic displacement parameters (Å^2^)

**Table:**

	*x*	*y*	*z*	*U*_iso_*/*U*_eq_	
Zn1	−0.06451 (2)	0.622158 (11)	0.714672 (8)	0.03146 (7)	
O1	−0.21883 (16)	0.54529 (7)	0.74818 (6)	0.0431 (3)	
O2	0.00514 (16)	0.45411 (8)	0.76070 (7)	0.0509 (4)	
O3	−0.79388 (15)	0.22499 (7)	0.79980 (6)	0.0421 (3)	
O4	−0.58272 (17)	0.16416 (8)	0.86719 (6)	0.0474 (3)	
O5	0.9256 (2)	0.74280 (12)	0.91104 (8)	0.0654 (5)	
O6	0.3534 (2)	0.50254 (9)	0.39196 (7)	0.0525 (4)	
O7	0.4650 (4)	0.24025 (15)	0.98388 (10)	0.0887 (7)	
N1	0.15225 (17)	0.66275 (8)	0.77281 (6)	0.0339 (3)	
N2	0.7742 (2)	0.70378 (11)	0.88055 (7)	0.0503 (4)	
N3	0.02911 (17)	0.59469 (8)	0.63468 (6)	0.0330 (3)	
N4	0.30194 (19)	0.49616 (9)	0.45026 (7)	0.0415 (4)	
N5	0.1156 (3)	0.60383 (14)	0.94885 (9)	0.0614 (5)	
N6	0.3640 (3)	0.30885 (14)	0.95959 (9)	0.0680 (5)	
C1	0.2999 (3)	0.61485 (10)	0.78018 (9)	0.0417 (4)	
C2	0.4614 (3)	0.64161 (11)	0.81024 (9)	0.0427 (4)	
C3	0.4744 (3)	0.72219 (11)	0.83570 (7)	0.0381 (4)	
C4	0.3214 (3)	0.77116 (11)	0.82951 (8)	0.0407 (4)	
C5	0.1641 (3)	0.74000 (10)	0.79765 (8)	0.0376 (4)	
C6	0.6445 (3)	0.75430 (13)	0.86763 (9)	0.0491 (5)	
C7	−0.0049 (3)	0.65017 (10)	0.58845 (8)	0.0369 (4)	
C8	0.0624 (3)	0.64167 (11)	0.53383 (8)	0.0409 (4)	
C9	0.1692 (3)	0.57299 (10)	0.52502 (8)	0.0376 (4)	
C10	0.2016 (3)	0.51452 (11)	0.57240 (8)	0.0414 (4)	
C11	0.1296 (3)	0.52711 (11)	0.62583 (8)	0.0405 (4)	
C12	0.2375 (3)	0.56456 (12)	0.46607 (9)	0.0474 (5)	
C13	−0.2764 (2)	0.41148 (9)	0.78608 (7)	0.0318 (3)	
C14	−0.4586 (2)	0.42012 (9)	0.76680 (8)	0.0356 (4)	
C15	−0.5772 (2)	0.36017 (10)	0.78215 (8)	0.0357 (4)	
C16	−0.5140 (2)	0.29122 (9)	0.81793 (7)	0.0314 (3)	
C17	−0.3329 (2)	0.28438 (10)	0.83923 (8)	0.0363 (4)	
C18	−0.2141 (2)	0.34347 (10)	0.82278 (8)	0.0359 (4)	
C19	−0.1499 (2)	0.47331 (10)	0.76378 (7)	0.0344 (4)	
C20	−0.6376 (2)	0.22133 (9)	0.83015 (8)	0.0348 (4)	
C21	0.1206 (3)	0.54202 (17)	0.90740 (10)	0.0629 (6)	
C22	0.2009 (3)	0.46555 (16)	0.92026 (9)	0.0603 (6)	
C23	0.2809 (3)	0.44822 (15)	0.98042 (9)	0.0547 (5)	
C24	0.2726 (3)	0.51129 (17)	1.02396 (10)	0.0637 (6)	
C25	0.1912 (3)	0.58644 (18)	1.00653 (11)	0.0670 (7)	
C26	0.3730 (4)	0.36927 (16)	0.99751 (10)	0.0631 (6)	
H1	0.2921	0.5605	0.7640	0.0500*	
H2	0.5607	0.6065	0.8136	0.0512*	
H3	0.990 (6)	0.699 (3)	0.9271 (18)	0.131 (15)*	
H4	0.3247	0.8249	0.8468	0.0488*	
H5	0.0629	0.7739	0.7933	0.0451*	
H6	0.6572	0.8111	0.8783	0.0590*	
H7	−0.0770	0.6964	0.5934	0.0443*	
H8	0.0364	0.6819	0.5029	0.0490*	
H9	0.376 (4)	0.4540 (18)	0.3841 (12)	0.075 (9)*	
H10	0.2715	0.4672	0.5682	0.0497*	
H11	0.1514	0.4871	0.6571	0.0485*	
H12	0.2331	0.6109	0.4398	0.0568*	
H13	0.456 (5)	0.208 (3)	0.9572 (16)	0.105 (13)*	
H14	−0.5012	0.4668	0.7433	0.0427*	
H15	−0.6987	0.3660	0.7685	0.0429*	
H17	−0.2909	0.2396	0.8648	0.0436*	
H18	−0.0926	0.3376	0.8364	0.0431*	
H21	0.0658	0.5518	0.8672	0.0755*	
H22	0.2023	0.4255	0.8892	0.0723*	
H24	0.3222	0.5025	1.0649	0.0764*	
H25	0.1883	0.6278	1.0365	0.0804*	
H26	0.4388	0.3632	1.0366	0.0757*	

### Atomic displacement parameters (Å^2^)

**Table:**

	*U*^11^	*U*^22^	*U*^33^	*U*^12^	*U*^13^	*U*^23^
Zn1	0.02747 (10)	0.02856 (10)	0.03936 (11)	0.00125 (6)	0.00831 (7)	0.00241 (7)
O1	0.0429 (7)	0.0306 (6)	0.0598 (8)	−0.0038 (5)	0.0205 (6)	0.0039 (5)
O2	0.0326 (6)	0.0496 (8)	0.0730 (9)	−0.0027 (6)	0.0165 (6)	0.0068 (7)
O3	0.0330 (6)	0.0317 (6)	0.0612 (8)	−0.0067 (5)	0.0050 (6)	0.0005 (5)
O4	0.0488 (7)	0.0361 (7)	0.0571 (8)	−0.0058 (6)	0.0077 (6)	0.0126 (6)
O5	0.0478 (8)	0.0793 (12)	0.0652 (10)	−0.0185 (8)	−0.0049 (7)	−0.0220 (9)
O6	0.0630 (9)	0.0467 (8)	0.0536 (8)	−0.0017 (7)	0.0281 (7)	−0.0102 (6)
O7	0.1144 (17)	0.0847 (14)	0.0620 (12)	0.0132 (12)	−0.0030 (11)	0.0018 (11)
N1	0.0327 (7)	0.0311 (7)	0.0382 (7)	0.0005 (5)	0.0066 (6)	0.0001 (6)
N2	0.0416 (9)	0.0650 (11)	0.0428 (9)	−0.0147 (8)	0.0011 (7)	−0.0138 (8)
N3	0.0293 (7)	0.0324 (7)	0.0377 (7)	−0.0008 (5)	0.0062 (6)	−0.0013 (6)
N4	0.0367 (8)	0.0466 (8)	0.0422 (8)	−0.0037 (6)	0.0091 (6)	−0.0074 (7)
N5	0.0432 (9)	0.0849 (14)	0.0554 (11)	−0.0078 (9)	0.0050 (8)	−0.0060 (10)
N6	0.0698 (13)	0.0799 (14)	0.0540 (11)	−0.0014 (11)	0.0079 (9)	0.0014 (10)
C1	0.0360 (9)	0.0327 (8)	0.0553 (11)	0.0017 (7)	0.0033 (8)	−0.0092 (7)
C2	0.0339 (9)	0.0408 (9)	0.0524 (11)	0.0025 (7)	0.0029 (8)	−0.0083 (8)
C3	0.0400 (9)	0.0413 (9)	0.0335 (8)	−0.0079 (7)	0.0069 (7)	−0.0032 (7)
C4	0.0525 (10)	0.0340 (8)	0.0365 (9)	−0.0024 (7)	0.0098 (8)	−0.0069 (7)
C5	0.0423 (9)	0.0345 (8)	0.0381 (9)	0.0054 (7)	0.0129 (7)	−0.0010 (7)
C6	0.0511 (11)	0.0491 (11)	0.0462 (11)	−0.0129 (9)	0.0037 (9)	−0.0101 (8)
C7	0.0375 (9)	0.0322 (8)	0.0410 (9)	0.0028 (7)	0.0061 (7)	−0.0008 (7)
C8	0.0492 (10)	0.0351 (8)	0.0388 (9)	0.0023 (7)	0.0081 (8)	0.0037 (7)
C9	0.0370 (9)	0.0373 (9)	0.0394 (9)	−0.0036 (7)	0.0086 (7)	−0.0043 (7)
C10	0.0399 (9)	0.0395 (9)	0.0454 (10)	0.0090 (7)	0.0083 (8)	−0.0012 (7)
C11	0.0413 (9)	0.0385 (9)	0.0416 (9)	0.0070 (7)	0.0063 (7)	0.0046 (7)
C12	0.0593 (12)	0.0405 (10)	0.0461 (10)	−0.0002 (8)	0.0204 (9)	−0.0002 (8)
C13	0.0309 (8)	0.0272 (7)	0.0385 (8)	−0.0034 (6)	0.0094 (6)	−0.0041 (6)
C14	0.0328 (8)	0.0256 (7)	0.0491 (9)	0.0022 (6)	0.0085 (7)	0.0036 (7)
C15	0.0274 (8)	0.0311 (8)	0.0490 (10)	0.0002 (6)	0.0066 (7)	0.0018 (7)
C16	0.0313 (8)	0.0270 (7)	0.0371 (8)	−0.0027 (6)	0.0085 (6)	−0.0013 (6)
C17	0.0348 (8)	0.0334 (8)	0.0402 (9)	0.0005 (6)	0.0037 (7)	0.0061 (7)
C18	0.0287 (8)	0.0367 (8)	0.0417 (9)	−0.0014 (6)	0.0029 (7)	0.0006 (7)
C19	0.0357 (8)	0.0313 (8)	0.0373 (8)	−0.0059 (6)	0.0091 (7)	−0.0045 (6)
C20	0.0351 (8)	0.0279 (8)	0.0431 (9)	−0.0031 (6)	0.0111 (7)	−0.0022 (7)
C21	0.0549 (12)	0.0891 (17)	0.0432 (11)	−0.0147 (12)	0.0025 (9)	−0.0012 (11)
C22	0.0608 (13)	0.0814 (16)	0.0392 (10)	−0.0156 (12)	0.0096 (9)	−0.0062 (10)
C23	0.0451 (11)	0.0778 (14)	0.0426 (10)	−0.0173 (10)	0.0114 (8)	−0.0015 (10)
C24	0.0549 (13)	0.0956 (18)	0.0397 (11)	−0.0098 (12)	0.0045 (9)	−0.0071 (11)
C25	0.0539 (13)	0.0950 (19)	0.0515 (13)	−0.0069 (13)	0.0057 (10)	−0.0186 (12)
C26	0.0637 (14)	0.0843 (17)	0.0420 (11)	−0.0127 (12)	0.0102 (10)	0.0050 (11)

### Geometric parameters (Å, º)

**Table:**

Zn1—O1	1.9088 (14)	C14—C15	1.383 (3)
Zn1—O3^i^	1.9501 (13)	C15—C16	1.387 (3)
Zn1—N1	2.0289 (13)	C16—C17	1.388 (3)
Zn1—N3	2.0327 (15)	C16—C20	1.500 (3)
O1—C19	1.279 (2)	C17—C18	1.383 (3)
O2—C19	1.226 (2)	C21—C22	1.366 (4)
O3—C20	1.2713 (19)	C22—C23	1.394 (3)
O4—C20	1.245 (2)	C23—C24	1.389 (4)
O5—N2	1.385 (3)	C23—C26	1.453 (4)
O6—N4	1.393 (3)	C24—C25	1.368 (4)
O7—N6	1.388 (4)	O5—H3	0.89 (4)
N1—C1	1.342 (3)	O6—H9	0.81 (3)
N1—C5	1.336 (2)	O7—H13	0.78 (4)
N2—C6	1.267 (3)	C1—H1	0.930
N3—C7	1.336 (3)	C2—H2	0.930
N3—C11	1.344 (3)	C4—H4	0.930
N4—C12	1.258 (3)	C5—H5	0.930
N5—C21	1.339 (4)	C6—H6	0.930
N5—C25	1.337 (3)	C7—H7	0.930
N6—C26	1.262 (4)	C8—H8	0.930
C1—C2	1.369 (3)	C10—H10	0.930
C2—C3	1.390 (3)	C11—H11	0.930
C3—C4	1.385 (3)	C12—H12	0.930
C3—C6	1.464 (3)	C14—H14	0.930
C4—C5	1.381 (3)	C15—H15	0.930
C7—C8	1.375 (3)	C17—H17	0.930
C8—C9	1.386 (3)	C18—H18	0.930
C9—C10	1.385 (3)	C21—H21	0.930
C9—C12	1.467 (3)	C22—H22	0.930
C10—C11	1.378 (3)	C24—H24	0.930
C13—C14	1.390 (3)	C25—H25	0.930
C13—C18	1.384 (3)	C26—H26	0.930
C13—C19	1.502 (3)		
			
O1—Zn1—O3^i^	103.92 (6)	N5—C21—C22	124.4 (2)
O1—Zn1—N1	116.51 (6)	C21—C22—C23	119.2 (2)
O1—Zn1—N3	120.49 (6)	C22—C23—C24	116.8 (3)
O3^i^—Zn1—N1	102.30 (6)	C22—C23—C26	122.5 (2)
O3^i^—Zn1—N3	107.02 (6)	C24—C23—C26	120.69 (19)
N1—Zn1—N3	104.77 (6)	C23—C24—C25	119.9 (2)
Zn1—O1—C19	114.91 (12)	N5—C25—C24	123.7 (3)
Zn1^ii^—O3—C20	119.68 (10)	N6—C26—C23	120.4 (2)
Zn1—N1—C1	118.72 (11)	N2—O5—H3	102 (3)
Zn1—N1—C5	123.01 (11)	N4—O6—H9	103 (2)
C1—N1—C5	117.62 (14)	N6—O7—H13	104 (3)
O5—N2—C6	112.76 (18)	N1—C1—H1	118.183
Zn1—N3—C7	117.37 (12)	C2—C1—H1	118.181
Zn1—N3—C11	124.92 (11)	C1—C2—H2	120.577
C7—N3—C11	117.63 (15)	C3—C2—H2	120.575
O6—N4—C12	111.28 (16)	C3—C4—H4	120.076
C21—N5—C25	116.0 (3)	C5—C4—H4	120.063
O7—N6—C26	111.6 (2)	N1—C5—H5	118.876
N1—C1—C2	123.64 (16)	C4—C5—H5	118.881
C1—C2—C3	118.85 (16)	N2—C6—H6	120.410
C2—C3—C4	117.76 (16)	C3—C6—H6	120.420
C2—C3—C6	121.03 (17)	N3—C7—H7	118.651
C4—C3—C6	121.21 (17)	C8—C7—H7	118.656
C3—C4—C5	119.86 (16)	C7—C8—H8	120.074
N1—C5—C4	122.24 (16)	C9—C8—H8	120.082
N2—C6—C3	119.17 (18)	C9—C10—H10	120.301
N3—C7—C8	122.69 (16)	C11—C10—H10	120.286
C7—C8—C9	119.84 (16)	N3—C11—H11	118.577
C8—C9—C10	117.54 (17)	C10—C11—H11	118.577
C8—C9—C12	118.92 (16)	N4—C12—H12	119.234
C10—C9—C12	123.51 (16)	C9—C12—H12	119.230
C9—C10—C11	119.41 (17)	C13—C14—H14	119.625
N3—C11—C10	122.85 (16)	C15—C14—H14	119.627
N4—C12—C9	121.54 (18)	C14—C15—H15	120.158
C14—C13—C18	119.43 (15)	C16—C15—H15	120.177
C14—C13—C19	119.41 (14)	C16—C17—H17	119.676
C18—C13—C19	121.06 (14)	C18—C17—H17	119.670
C13—C14—C15	120.75 (14)	C13—C18—H18	120.059
C14—C15—C16	119.66 (15)	C17—C18—H18	120.071
C15—C16—C17	119.55 (15)	N5—C21—H21	117.814
C15—C16—C20	120.58 (14)	C22—C21—H21	117.818
C17—C16—C20	119.74 (14)	C21—C22—H22	120.395
C16—C17—C18	120.65 (15)	C23—C22—H22	120.411
C13—C18—C17	119.87 (15)	C23—C24—H24	120.064
O1—C19—O2	124.31 (16)	C25—C24—H24	120.069
O1—C19—C13	114.32 (14)	N5—C25—H25	118.127
O2—C19—C13	121.36 (15)	C24—C25—H25	118.128
O3—C20—O4	124.72 (15)	N6—C26—H26	119.823
O3—C20—C16	115.58 (14)	C23—C26—H26	119.820
O4—C20—C16	119.68 (14)		
			
O1—Zn1—O3^i^—C20^i^	−51.57 (11)	C2—C3—C6—N2	−11.0 (3)
O3^i^—Zn1—O1—C19	−177.30 (8)	C4—C3—C6—N2	169.38 (16)
O1—Zn1—N1—C1	84.68 (11)	C6—C3—C4—C5	178.20 (15)
O1—Zn1—N1—C5	−104.80 (11)	C3—C4—C5—N1	1.0 (3)
N1—Zn1—O1—C19	−65.65 (10)	N3—C7—C8—C9	0.5 (3)
O1—Zn1—N3—C7	122.99 (9)	C7—C8—C9—C10	0.8 (3)
O1—Zn1—N3—C11	−60.34 (11)	C7—C8—C9—C12	179.15 (14)
N3—Zn1—O1—C19	62.98 (10)	C8—C9—C10—C11	−0.7 (3)
O3^i^—Zn1—N1—C1	−162.75 (9)	C8—C9—C12—N4	−164.73 (16)
O3^i^—Zn1—N1—C5	7.77 (12)	C10—C9—C12—N4	13.5 (3)
N1—Zn1—O3^i^—C20^i^	−173.22 (10)	C12—C9—C10—C11	−179.00 (15)
O3^i^—Zn1—N3—C7	4.81 (10)	C9—C10—C11—N3	−0.6 (3)
O3^i^—Zn1—N3—C11	−178.51 (9)	C14—C13—C18—C17	0.8 (3)
N3—Zn1—O3^i^—C20^i^	76.92 (11)	C18—C13—C14—C15	−2.2 (3)
N1—Zn1—N3—C7	−103.31 (9)	C14—C13—C19—O1	25.0 (2)
N1—Zn1—N3—C11	73.36 (11)	C14—C13—C19—O2	−154.27 (14)
N3—Zn1—N1—C1	−51.20 (10)	C19—C13—C14—C15	174.36 (13)
N3—Zn1—N1—C5	119.32 (10)	C18—C13—C19—O1	−158.55 (14)
Zn1—O1—C19—O2	3.1 (2)	C18—C13—C19—O2	22.2 (3)
Zn1—O1—C19—C13	−176.10 (8)	C19—C13—C18—C17	−175.65 (13)
Zn1^ii^—O3—C20—O4	−22.5 (3)	C13—C14—C15—C16	0.9 (3)
Zn1^ii^—O3—C20—C16	156.16 (9)	C14—C15—C16—C17	1.7 (3)
Zn1—N1—C1—C2	169.39 (12)	C14—C15—C16—C20	−174.19 (14)
Zn1—N1—C5—C4	−170.06 (10)	C15—C16—C17—C18	−3.0 (3)
C1—N1—C5—C4	0.6 (3)	C15—C16—C20—O3	8.3 (3)
C5—N1—C1—C2	−1.6 (3)	C15—C16—C20—O4	−172.96 (15)
O5—N2—C6—C3	−179.56 (15)	C17—C16—C20—O3	−167.49 (14)
Zn1—N3—C7—C8	175.10 (10)	C17—C16—C20—O4	11.2 (3)
Zn1—N3—C11—C10	−174.78 (10)	C20—C16—C17—C18	172.87 (14)
C7—N3—C11—C10	1.9 (3)	C16—C17—C18—C13	1.8 (3)
C11—N3—C7—C8	−1.8 (3)	N5—C21—C22—C23	1.4 (4)
O6—N4—C12—C9	178.33 (14)	C21—C22—C23—C24	0.2 (4)
C21—N5—C25—C24	0.9 (4)	C21—C22—C23—C26	−178.39 (19)
C25—N5—C21—C22	−1.9 (4)	C22—C23—C24—C25	−1.0 (4)
O7—N6—C26—C23	178.2 (2)	C22—C23—C26—N6	−9.1 (4)
N1—C1—C2—C3	1.1 (3)	C24—C23—C26—N6	172.4 (2)
C1—C2—C3—C4	0.4 (3)	C26—C23—C24—C25	177.53 (19)
C1—C2—C3—C6	−179.20 (16)	C23—C24—C25—N5	0.5 (4)
C2—C3—C4—C5	−1.4 (3)		

Symmetry codes: (i) −*x*−1, *y*+1/2, −*z*+3/2; (ii) −*x*−1, *y*−1/2, −*z*+3/2.

### Hydrogen-bond geometry (Å, º)

**Table:**

*D*—H···*A*	*D*—H	H···*A*	*D*···*A*	*D*—H···*A*
O5—H3···N5^iii^	0.89 (5)	1.81 (4)	2.692 (3)	172 (4)
O6—H9···O4^iv^	0.81 (3)	1.94 (3)	2.752 (3)	177 (3)
O7—H13···O4^iii^	0.77 (4)	2.07 (4)	2.800 (3)	158 (4)

Symmetry codes: (iii) *x*+1, *y*, *z*; (iv) *x*+1, −*y*+1/2, *z*−1/2.
